# THE RISE OF LONELINESS: TRENDS AMONG A LARGE MEDICARE ADVANTAGE COHORT OVER THE COVID-19 PANDEMIC

**DOI:** 10.1093/geroni/igac059.2923

**Published:** 2022-12-20

**Authors:** Kelsey McNamara, Ellen Rudy

**Affiliations:** Papa Inc., Miami, Florida, United States; Papa Inc., Miami, Florida, United States

## Abstract

Loneliness is an established risk factor for premature mortality, especially among older adults. National trends are indicating an increase in loneliness after the COVID-19 pandemic. We sought to explore annual trends in loneliness and quality of life among a large dataset of Medicare Advantage (MA) members. Papa Inc. is a national service that pairs older adults with “Papa Pals” who provide companionship and assistance with everyday tasks. Participants have free access if their health plan offers it. Analysis included members from MA plans that were active with Papa January 2020 through March 2022. Members (n=11,037) were assessed at enrollment screening (UCLA Three-Item Loneliness Scale, CDC’s Healthy Days Measure). Analysis used Chi-square tests, significance set at p < 0.05. Among the sample, 81% were aged 65+, 14% were from a majority non-white community. Analysis revealed the following trends in annual prevalence rates (2020; 2021; 2022): lonely (43.2%; 39.3%; 40.9%), severely lonely (9.2%; 12.1%; 15.1%), 14+ physically unhealthy days (24.3%; 19.9%; 39.3%). In regression analysis, loneliness score increased by 0.05 (95CI: 0.02 - 0.07) per quarter over the 27 months. Our data mimics other sources highlighting a slow and steady increase in loneliness suggesting older adults’ hesitancy to engage in “normal activities.” Changes in unhealthy days follow COVID-19 trends with a significant increase during the height of the Omicron variant. The prolonged effects of isolation and loneliness are profound, thus greater emphasis should be placed on population level interventions, especially those connected to the healthcare system, to help address loneliness.

